# Regulatory principles and mechanisms governing the onset of random X-chromosome inactivation

**DOI:** 10.1016/j.gde.2023.102063

**Published:** 2023-08

**Authors:** Till Schwämmle, Edda G Schulz

**Affiliations:** Otto Warburg Laboratories, Max Planck Institute for Molecular Genetics, Berlin, Germany

## Abstract

X-chromosome inactivation (XCI) has evolved in mammals to compensate for the difference in X-chromosomal dosage between the sexes. In placental mammals, XCI is initiated during early embryonic development through upregulation of the long noncoding RNA Xist from one randomly chosen X chromosome in each female cell. The Xist locus must thus integrate both X-linked and developmental trans-regulatory factors in a dosage-dependent manner. Furthermore, the two alleles must coordinate to ensure inactivation of exactly one X chromosome per cell. In this review, we summarize the regulatory principles that govern the onset of XCI. We go on to provide an overview over the factors that have been implicated in Xist regulation and discuss recent advances in our understanding of how Xist’s cis-regulatory landscape integrates information in a precise fashion.

## Highlights

• Complex regulatory interactions ensure female-specific monoallelic Xist expression.
• Stochastic upregulation combined with a negative feedback can explain the Xist pattern.
• A series of transcription factors activate Xist in different cellular context.
• Differentiation cues control distal enhancers, X-dosage is sensed promoter-proximally.

**Current Opinion in Genetics & Development** 2023, **81**:102063

This review comes from a themed issue on **Genome Architecture and Expression**

Edited by **Giacomo Cavalli** and **Job Dekker**

For complete overview of the section, please refer to the article collection, “Genome Architecture and Expression (2023)”

https://doi.org/10.1016/j.gde.2023.102063

0959–437X/© 2023 The Author(s). Published by Elsevier Ltd. This is an open access article under the CC BY license (http://creativecommons.org/licenses/by/4.0/)

## Introduction

The sexual dimorphism of mammals is encoded in the genome through sex chromosomes. While males carry a single X and a gene-poor Y chromosome, primarily needed for sex determination, females develop in the presence of two X chromosomes. The X chromosome contains a wealth of genetic information that is largely unrelated to sex-specific phenotypes. In order to avoid a harmful double amount of X-linked gene products, female mammals have developed X-chromosome inactivation (XCI) as a strategy for dosage compensation between the sexes (recently reviewed in [1]). In placental mammals, XCI results in near-complete silencing of one randomly chosen X chromosome in each female cell.

XCI is established during early embryonic development and inherited through all subsequent cell divisions. It is initiated by the upregulation of the long noncoding RNA (lncRNA) Xist, which is encoded on the X chromosome. Between 50 and 200 Xist molecules spread over the entire chromosome 2, 3••. They form a repressive compartment by nucleating RNA–protein assemblies that contain 1–2 Xist RNA copies and a suprastoichiometric amount of associated proteins, including the essential silencing factor SPEN(SHARP) 2, 3••, 4, 5, 6.

**Figure 1:**
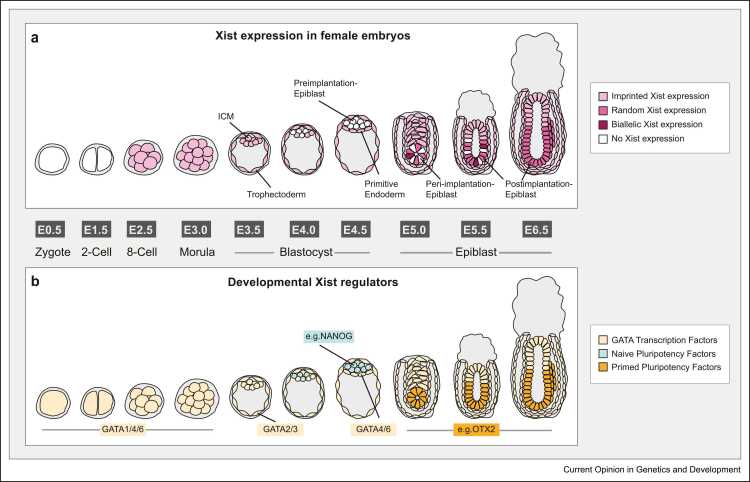
Developmental control of Xist expression. The first 6.5 days of mouse development are shown together with the associated Xist expression state observed in females **(a)** and the expression pattern of putative Xist regulators **(b)**. The embryonic day and developmental stage are indicated between the two panels. Imprinted Xist expression (light pink) is initiated shortly before the 4-cell stage by several GATA transcription factors as indicated and later maintained in all extraembryonic lineages (light yellow). Downregulation of GATA factors and upregulation of naive pluripotency factors, such as NANOG, PRDM14, REX1, and ESRRB (teal), lead to Xist downregulation in the preimplantation epiblast, which will give rise to the embryonic lineages. Around the time of implantation into the uterus, random Xist upregulation is initiated by primed pluripotency factors, such as OTX2 and SMAD2/3 (orange), in the epiblast cells, where some cells initially upregulate Xist from both alleles (dark purple) before reaching the random monoallelic state (pink). Xist expression patterns are based on 10, 11.

In mice, XCI occurs in two waves (Figure 1): Xist is first upregulated at the 2–4-cell stage, where its monoallelic expression is imprinted through a maternal H3K27me3 domain deposited in oocytes, resulting in the silencing of the paternal X in all cells [7]. This rodent-specific imprinted version of XCI (iXCI) persists in extraembryonic tissues, but is reversed in the pluripotent cells of the late blastocyst, where Xist is downregulated [8]. Shortly after, random XCI (rXCI) is initiated in the peri-implantation epiblast. During a 2-day time window, cells transition from a Xist-negative state at embryonic day 4.5 (E4.5) to a monoallelic state in 100% epiblast cells at E6.5 with transitioning through a phase, where Xist-negative, mono-, and even biallelic cells coexist (Figure 1a) 9, 10, 11, 12. Murine embryonic stem cells (mESCs), which are derived from the blastocyst stage, are a powerful cell culture model to study the onset of rXCI. Female mESCs carry two active X chromosomes and will initiate rXCI upon differentiation. A variety of differentiation protocols have been used to investigate XCI onset, which vary significantly in their efficiency of Xist upregulation (in some cases requiring more than a week to reach>50% Xist-positive cells) 13, 14, 15, which has potentially resulted in conflicting results. More recently adopted protocols, which start from a naive pluripotent state (2i medium), appear to more closely resemble the *in vivo* dynamics of Xist upregulation, reaching 50–80% of Xist-positive cells after 2 days with a transient occurrence of biallelic Xist expression in a subset of cells 12, 13, 16.

While biallelic Xist expression is transient in mice, in primates, including humans, XIST is expressed from both X chromosomes for an extended period of time before it transitions to a monoallelic state after implantation 17, 18. This remarkable expression pattern of Xist/XIST presents multiple conceptual challenges regarding its regulation: how does the cell sense the number of X chromosomes to ensure female specificity? Which mechanisms break the symmetry between the two X chromosomes at the correct developmental time and drive the choice of the future inactive X (Xi)? What epigenetic processes allow the cells to memorize the inactivated allele? In the following sections, we will review recent advances in our conceptual understanding of this process and of the molecular players that govern it. Considering that the cellular events underlying XCI initiation have mostly been investigated in the mouse, this system will be the focus of our review.

## The stochastic model of X-chromosome inactivation onset

**Figure 2:**
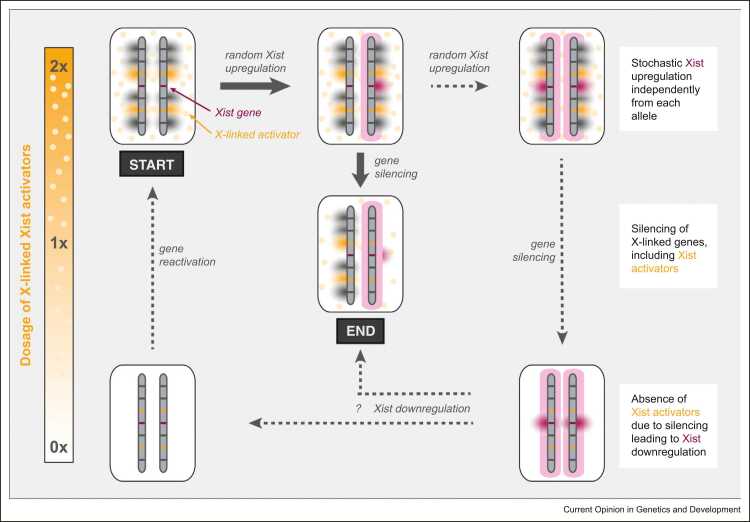
The stochastic model of XCI onset. The initial state before the onset of rXCI, where both X chromosomes are active in females, is shown in the upper-left corner (START) and the final state in the center (END). The panels are ordered according to the dosage of X-linked activators. Thus, panels with a 2x dose are located in the top row, with a 1x dose in the middle row and with a 0x dose in the bottom row. Vertical bars indicate the two X chromosomes, where Xist activators (orange) and other X-linked genes (dark gray) are active on both alleles. The small, horizontal bars represent X-chromosomal genes, with *Xist* colored in purple and X-linked activators colored in orange. The small clouds next to the genes represent active transcription. Upon differentiation, a double dose (upper row) of X-linked activators (orange circles) allows stochastic upregulation of Xist (upper middle). Xist clouds are pictured as large pink shading around the entire chromosome. Xist-induced chromosome-wide gene silencing will reduce the activator dose, thus preventing Xist upregulation from the second allele and thereby stabilizing the monoallelic state (middle, END). In a fraction of cells, Xist will be upregulated from the second allele before the activator is silenced, leading to biallelic Xist expression (upper right). Silencing of both copies of essential Xist activators (lower right) will lead to Xist downregulation either on one (center) or on both alleles (lower left), thereby destabilizing the biallelic state.

Initially, it was assumed that the processes of sensing the number of X chromosomes in the nucleus and choosing one for inactivation were distinct phenomena [19]. However, it has become increasingly clear that both are emerging properties of a complex interplay of cis- and trans-acting mechanisms (as reviewed in [20]). Initial upregulation of Xist appears to occur independently on each allele, which is supported by the fact that expression from both alleles is observed in a subset of cells at the onset of rXCI *in vitro* and *in vivo* 10, 11, 12, 13, 16. Coordination between the two X chromosomes before Xist upregulation had been hypothesized (e.g. through chromosome pairing), but has been largely disproven 21, 22, 23. Instead, it has been shown that Xist is upregulated stochastically, and that monoallelic expression is ensured through a feedback mechanism once XCI has been initiated (Figure 2) [24].

In the stochastic model, the probability to upregulate Xist is modulated by trans-acting X-linked Xist activators 24, 25. By promoting *Xist* upregulation in an X-dosage-dependent manner, it contributes to female specificity providing an explanation why Xist is upregulated only in cells with two X chromosomes. For cells to be able to reliably distinguish between a single and a double dose of X-linked activators, an activation threshold has been proposed that can only be reached, if two or more X chromosomes are present [24]. Once XCI has been initiated on one chromosome, the proposed X-linked activators are silenced *in cis*, causing their levels to drop below the activation threshold, and thereby preventing Xist upregulation from the second X chromosome. If all X chromosomes in a cell initiate XCI (i.e. upregulation in male cells or biallelic Xist in females), complete silencing of essential X-linked activators would lead to Xist downregulation from one or both alleles (Figure 2), which we have previously observed experimentally [11]. Such a negative feedback would ensure that at least one X remains active in each cell. This model can explain the presence of a single active X in X-aneuploidies, such as Klinefelter (XXY) and Turner syndrome (XO), as well as the presence of two active X’s in female tetraploid cells, when assuming an ∼2-fold dilution of X-linked activators due to ∼2-fold increase in nuclear volume [11].

In the stochastic model, X-linked activators are predicted to control Xist in a dose-dependent manner *in trans* and to be silenced rapidly. Global analysis of X-linked silencing dynamics revealed that silencing can occur as early as 4–6 h after Xist upregulation [26], which would potentially allow cells to undergo two attempts to reach the monoallelic state within the 2-day time window of XCI onset. In an alternative, not mutually exclusive scenario, an X-linked activator might not be subject to XCI, but rather escape inactivation [27]. Such an escaping activator would allow female-specific maintenance of Xist expression through persistent high activator levels, but would not allow feedback control, which is required to ensure monoallelic expression. We have recently shown through mathematical modeling that stochastic Xist upregulation controlled by a silenced X-linked activator can reproduce female-specific and monoallelic upregulation, when assuming epigenetic memory of the Xist expression state through a *cis*-acting positive feedback loop [11]. Various aspects of this model are supported through experimental evidence (as reviewed in [20]). However, the underlying molecular events are only starting to be elucidated.

## Trans-acting Xist regulators

The stochastic model of XCI requires tight transcriptional control of Xist to ensure female specificity through X-dosage-dependent regulation, but also to ensure a sufficiently slow rate of Xist upregulation required for symmetry-breaking to reach a monoallelic expression state.

### X-dosage-dependent regulation

**Figure 3:**
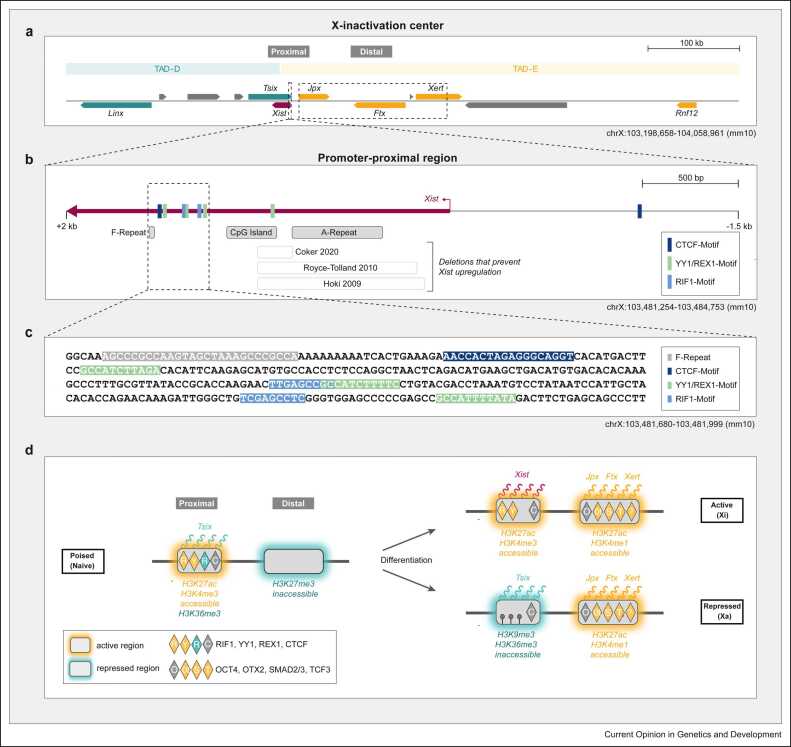
Regulation of distal and promoter-proximal cis-regulatory elements at the onset of rXCI. **(a)** The genomic region surrounding the *Xist* gene (purple). Xist regulators are colored in orange (activators) and teal (repressors). The promoter-proximal and distal regulatory regions are indicated as dashed boxes. **(b)** Detailed view of the promoter-proximal area. Binding motifs of Xist regulators are shown as small vertical bars and indicated in the zoom-in below in **(c)**. The CpG island of the *Xist* promoter and regions encoding the repeats A (responsible for Xist RNA-mediated silencing) and F are indicated. Regions that, when deleted, prevent Xist upregulation are depicted as boxes with gray outlines together with the relevant references. **(d)** The *Xist* locus assumes three different states. While distal regions are repressed in the naive state, they are activated upon differentiation, bind several transcription factors (OTX2, SMAD2/3, and TCF3) associated with epiblast differentiation, and transcribe several lncRNAs (Jpx, Ftx, Xert, and wiggled lines). The promoter-proximal region is in a poised state in naive cells (repressive antisense transcription by Tsix leading to H3K36me3 deposition, bound by repressor REX1, and activators YY1 and RIF1). Upon differentiation, the active X (Xa) assumes a repressed state associated with H3K9me3 and DNA methylation (black lollipops), while the Xi assumes an active state leading to Xist expression. Repressive and activating elements and regulators are colored in teal and orange, respectively. The RIF1 motif was identified in [46].

X-dosage-sensitive regulation is most likely provided by multiple genes, some of which remain to be discovered. The best-studied X-linked Xist activator is the E3 ubiquitin ligase RLIM (Rnf12), which is encoded close to the *Xist* gene (Figure 3a) and silenced rapidly upon Xist upregulation as predicted by the stochastic model 16, 28. It activates Xist by targeting the Xist repressor REX1 for degradation 28, 29. Heterozygous *Rnf12* mutant cells (that carry a single *Rnf12* dose such as male cells) can however still upregulate Xist [28]. *In vivo*, a complete absence of RNF12 prevents Xist expression during iXCI, but not during rXCI 30, 31, 32. *In vitro,* it appears to depend on the differentiation system whether RNF12 is essential for the onset of XCI 14, 33. Decreased O_2_ concentrations, which generally enhance Xist expression, reduce the dependency of XCI on RNF12 [14]. Another gene implicated in X-dosage-sensitive Xist regulation is the lncRNA *Jpx*, which is located just upstream of the *Xist* gene (Figure 3a) and escapes XCI 27, 34. Using a suboptimal differentiation protocol, deletion of one *Jpx* copy in female mESCs had initially been reported to prevent Xist upregulation [34]. Other studies in more efficient differentiation systems and in embryos *in vivo* could however not confirm these findings 21, 35••, 36. Even a combinatorial, heterozygous deletion of *Jpx* and *Rnf12* does not abrogate Xist expression [21], suggesting that important X-linked regulators remain to be discovered. The X-linked H3K4me3-demethylase KDM5C was recently described as a potential candidate, as its absence results in somewhat reduced Xist levels in differentiating mESCs and female-specific lethality *in vivo* [37]. However, female-specific lethality might not necessarily be due to defective XCI, but rather due to other functions of the gene, which might be taken over by the Y-homolog KDM5D in males. Since Kdm5c and Jpx escape XCI and are thus unaffected by Xist expression, they could only contribute to female-specific Xist expression, but could not participate in the predicted negative feedback to ensure monoallelic expression (see above) 27, 37.

### Developmental regulation

In female mice, Xist is almost universally expressed in developmental and somatic tissues, with the exception of pluripotent cells in the late blastocyst and the germline. Therefore, Xist expression was initially thought to be primarily governed through repression in those cells where it is absent. Accordingly, several pluripotency factors, such as OCT4, NANOG, REX1 (target of RNF12, see above), and PRDM14 38, 39, 40, have been shown to repress Xist. Although depletion of some of these factors in mESCs leads to Xist upregulation 38, 40, the deletion of their major binding site within the *Xist* locus (intron 1, see below) does not [41]. Therefore, Xist repression by pluripotency factors might be indirect through the prevention of cell differentiation. Indeed, several transcription factors associated with epiblast differentiation bind to Xist enhancer elements, including OTX2 and SMAD2/3 [35]. Interestingly, OTX2 binds to these enhancers in concert with OCT4 upon mESC differentiation 35••, 42. It thus remains to be seen whether OCT4 could also act as an activator of Xist, since it binds enhancer elements, or only functions as a repressor as previously suggested 38, 39.

It is becoming evident that Xist expression is activated and maintained by a diverse set of autosomal transcription factors in a tissue-specific manner. For example, P53 has been suggested to activate Xist during early brain development [43]. Moreover, we have recently identified the GATA transcription factor family as potent Xist activators, a subset of which drive initial Xist upregulation in preimplantation embryos [44]. The fact that different GATA factors are expressed in all cells of the early embryo, except in the pluripotent cells in the late blastocyst, where Xist is downregulated, again suggests that at this stage, the absence of activators contributes to Xist downregulation (Figure 1b).

### Other regulators

In recent years, a series of additional proteins have been implicated in Xist regulation. The ubiquitous transcription factor YY1 has been shown to function as a Xist activator [45]. Similarly, the absence of RIF1, which is known for its role in replication timing control, results in impaired Xist upregulation in differentiating mESCs 46•, 47. Furthermore, the epigenetic modifiers TRIM28/KAP1 and CHD8 have recently been implicated in Xist regulation 46•, 48. Another putative Xist regulator is the RNA-decapping enzyme DCP1A, suggested to control Xist’s repressive antisense transcript *Tsix* [49]. Finally, a new layer of control that affects Xist post-transcriptionally has recently been uncovered. Xist RNA stability appears to be controlled in a negative feedback loop, where loss of production stabilizes the RNA [3]. Interestingly, this mode of regulation has been linked to the SPEN protein, the key mediator of silencing during the initiation of XCI [6]. Intriguingly, loss of SPEN function leads to reduced XIST RNA stability 3••, 50, but to an increase in Xist production 3••, 4.

## The regulatory landscape around *Xist*

As summarized above, a variety of trans-acting factors involved in Xist regulation have been identified. But how are those signals sensed and interpreted by the *Xist* locus?

### The X-inactivation center

The *Xist* gene is located in the *X-inactivation center* (*Xic*), which contains a series of activating and repressive elements, including numerous regulatory lncRNA genes (Figure 3a). The *Xic* is split into two topologically associating domains (TADs), TAD-E and TAD-D [51], which contain elements that activate and repress Xist, respectively. *Xist* and its repressive antisense gene *Tsix* are transcribed across the TAD-D/-E boundary with the *Xist* promoter being located in TAD-E and the *Tsix* promoter in TAD-D. *Tsix* is transcribed through the entire *Xist* gene, including the promoter, and represses Xist *in cis* through depositing repressive histone modifications, such as H3K36me3 52•, 53, 54, 55.

Distal regulatory regions sense the developmental state of the cell, while promoter-proximal elements (Figure 3b–d) integrate information on X-chromosomal dosage [35]. While activity of TAD-D is reduced during differentiation, distal regulatory regions in TAD-E are activated, independent of whether the cell carries one or two X chromosomes (Figure 3d). In cells with one X chromosome, however, the promoter-proximal region of Xist is repressed, thus rendering the gene insensitive to activation by distal elements. A regulatory hierarchy between distal and proximal regulation thus allows integration of information on X-dosage and developmental time.

### Distal elements

The repressive effects of elements in TAD-D are thought to be largely mediated via regulation of Tsix, which allows communication across the TAD boundary via antisense transcription 56, 57, 58. Intriguingly, TAD-D contains another lncRNA locus, called *Linx*, which also represses Xist *in cis* but in a Tsix-independent manner, through a yet-unknown mechanism 51, 59•.

In TAD-E, numerous regulatory regions have been identified, including the three lncRNA genes *Jpx*, *Ftx*, and *Xert*, and an enhancer cluster within *Xert*, called *XertE*. They all activate Xist mostly *in cis* 34, 35••, 60, 61. These elements are located in the proximal half of the TAD, where a sub-TAD with increased contacts is formed upon differentiation [35]. The importance of long-range regulation for developmental control at the *Xic* is also supported by aberrant Xist upregulation upon inversions of the *Xist/Tsix* transcription unit or within TAD-D 62, 63.

Developmental control of distal regions in TAD-E appears to involve repression of the region in undifferentiated cells through a large H3K27me3 domain [64] and activation by differentiation-dependent transcription factors [35]. In addition, an element within intron 1 of *Xist*, which is bound by various pluripotency factors, has been suggested to repress Xist in undifferentiated cells [38]. However, deletion of the intron-1-binding site did not lead to premature Xist upregulation in naive cells [41]. The element might nevertheless act in a redundant fashion with distal regions.

### Promoter-proximal regulation

While distal elements are activated in both male and female cells, the promoter-proximal region appears to respond to X-dosage cues [35]. This region extends ∼2 kb from the transcription start site into the first exon of *Xist* (Figure 3b–c). It contains the somatic promoter P2 [65] and encodes the A repeat, which is required for Xist RNA-mediated silencing. This region is targeted by multiple trans-acting factors that have been implicated in X-dosage sensing (Figure 3b–c). The Xist repressor REX1, which is targeted by RNF12, competes with YY1 for several binding sites in the region 29, 45. Similarly, Jpx has been suggested to modulate binding of CTCF to this region and thereby affect contacts to distal elements 27, 34, 66•. The promoter-proximal region is indeed essential for Xist upregulation, since deletions in this area lead to complete loss of Xist expression 67, 68, 69. In naive cells, this region is in a ‘poised’ state and bound by YY1 and RIF1 35••, 45, 46•, but Xist expression is likely prevented by *Tsix* transcription in conjunction with lack of distal enhancer and lncRNA activity. Upon differentiation, the proximal region will maintain its active state on the Xi, where Xist is upregulated, and undergo heterochromatization through recruitment of H3K9me3 on the Xa [35], possibly mediated by *Tsix* transcription, TRIM28, and the G9A methyltransferase 46•, 52•, 70. Subsequently, a CpG island in the region will be asymmetrically methylated on the Xa, locking in the repressed state [71]. The promoter-proximal region thus appears to constitute a binary switch required to ensure female specificity and monoallelic Xist expression.

## Conclusions

In recent years, our understanding of developmental and tissue-specific Xist regulation has progressed significantly. As for other developmental genes, tissue specificity is largely controlled by long-range regulation. Near-ubiquitous expression in female cells is likely achieved via a diverse set of enhancers that are controlled by various lineage-specific transcription factors. By contrast, X-dosage sensing, choice of the X to inactivate, and establishment of epigenetic memory seem to be governed by a promoter-proximal regulatory element. This region acts as a binary switch that can assume a repressed or an active state, where the switching probability is determined by a complex interplay of different factors. How this bifunctional element is controlled, and whether it can indeed maintain an epigenetic memory, remains to be uncovered. Also, the search for trans-acting factors involved in X-dosage sensing will continue to eventually test whether the predicted activation threshold indeed exists and how it is implemented on the molecular level. Although much work has focussed on transcriptional control of Xist, the picture appears to be more complex. Feedback loops acting at the level of RNA stability fine-tune Xist levels, potentially to make sure that XCI stays restricted to the X chromosome. The mechanisms at play for post-transcriptional control of Xist will be an interesting topic for future studies.

## Declaration of Competing Interest

The authors declare no conflicts of interest.

## Data Availability

No data were used for the research described in the article.

## References

[1] Loda A., Collombet S., Heard E. (2022). Gene regulation in time and space during X-chromosome inactivation. Nat Rev Mol Cell Biol.

[2] Markaki Y., Gan Chong J., Wang Y., Jacobson E.C., Luong C., Tan S.Y.X., Jachowicz J.W., Strehle M., Maestrini D., Banerjee A.K. (2021). Xist nucleates local protein gradients to propagate silencing across the X chromosome. Cell.

[3••] Rodermund L., Coker H., Oldenkamp R., Wei G., Bowness J., Rajkumar B., Nesterova T., Susano Pinto D.M., Schermelleh L., Brockdorff N. (2021). Time-resolved structured illumination microscopy reveals key principles of Xist RNA spreading. Science.

[4] Jachowicz J.W., Strehle M., Banerjee A.K., Blanco M.R., Thai J., Guttman M. (2022). Xist spatially amplifies SHARP/SPEN recruitment to balance chromosome-wide silencing and specificity to the X chromosome. Nat Struct Mol Biol.

[5] Pandya-Jones A., Markaki Y., Serizay J., Chitiashvili T., Mancia Leon W.R., Damianov A., Chronis C., Papp B., Chen C.-K., McKee R. (2020). A protein assembly mediates Xist localization and gene silencing. Nature.

[6] Dossin F., Pinheiro I., Żylicz J.J., Roensch J., Collombet S., Le Saux A., Chelmicki T., Attia M., Kapoor V., Zhan Y. (2020). SPEN integrates transcriptional and epigenetic control of X-inactivation. Nature.

[7] Inoue A., Jiang L., Lu F., Zhang Y. (2017). Genomic imprinting of Xist by maternal H3K27me3. Genes Dev.

[8] Borensztein M., Okamoto I., Syx L., Guilbaud G., Picard C., Ancelin K., Galupa R., Diabangouaya P., Servant N., Barillot E. (2017). Contribution of epigenetic landscapes and transcription factors to X-chromosome reactivation in the inner cell mass. Nat Commun.

[9] Cheng S., Pei Y., He L., Peng G., Reinius B., Tam P.P.L., Jing N., Deng Q. (2019). Single-cell RNA-seq reveals cellular heterogeneity of pluripotency transition and X chromosome dynamics during early mouse development. Cell Rep.

[10] Shiura H., Abe K. (2019). Xist/Tsix expression dynamics during mouse peri-implantation development revealed by whole-mount 3D RNA-FISH. Sci Rep.

[11] Mutzel V., Okamoto I., Dunkel I., Saitou M., Giorgetti L., Heard E., Schulz E.G. (2019). A symmetric toggle switch explains the onset of random X inactivation in different mammals. Nat Struct Mol Biol.

[12] Sousa E.J., Stuart H.T., Bates L.E., Ghorbani M., Nichols J., Dietmann S., Silva J.C.R. (2018). Exit from naive pluripotency induces a transient X chromosome inactivation-like state in males. Cell Stem Cell.

[13] Guyochin A., Maenner S., Chu E.T.-J., Hentati A., Attia M., Avner P., Clerc P. (2014). Live cell imaging of the nascent inactive X chromosome during the early differentiation process of naive ES cells towards epiblast stem cells. PLoS One.

[14] Wang F., McCannell K.N., Bošković A., Zhu X., Shin J., Yu J., Gallant J., Byron M., Lawrence J.B., Zhu L.J. (2017). Rlim-dependent and -independent pathways for X chromosome inactivation in female ESCs. Cell Rep.

[15] Ahn J.Y., Lee J.T. (2010). Retinoic acid accelerates downregulation of the Xist repressor, Oct4, and increases the likelihood of Xist activation when Tsix is deficient. BMC Dev Biol.

[16] Pacini G., Dunkel I., Mages N., Mutzel V., Timmermann B., Marsico A., Schulz E.G. (2021). Integrated analysis of Xist upregulation and X-chromosome inactivation with single-cell and single-allele resolution. Nat Commun.

[17] Okamoto I., Nakamura T., Sasaki K., Yabuta Y., Iwatani C., Tsuchiya H., Nakamura S.-I., Ema M., Yamamoto T., Saitou M. (2021). The X chromosome dosage compensation program during the development of cynomolgus monkeys. Science.

[18] Okamoto I., Patrat C., Thépot D., Peynot N., Fauque P., Daniel N., Diabangouaya P., Wolf J.-P., Renard J.-P., Duranthon V. (2011). Eutherian mammals use diverse strategies to initiate X-chromosome inactivation during development. Nature.

[19] Rougeulle C., Avner P. (2003). Controlling X-inactivation in mammals: what does the centre hold?. Semin Cell Dev Biol.

[20] Mutzel V., Schulz E.G. (2020). Dosage sensing, threshold responses, and epigenetic memory: a systems biology perspective on random X-chromosome inactivation. Bioessays.

[21] Barakat T.S., Loos F., van Staveren S., Myronova E., Ghazvini M., Grootegoed J.A., Gribnau J. (2014). The trans-activator RNF12 and cis-acting elements effectuate X chromosome inactivation independent of X-pairing. Mol Cell.

[22] Pollex T., Heard E. (2019). Nuclear positioning and pairing of X-chromosome inactivation centers are not primary determinants during initiation of random X-inactivation. Nat Genet.

[23] Furlan G., Galupa R. (2022). Mechanisms of choice in X-chromosome inactivation. Cells.

[24] Monkhorst K., Jonkers I., Rentmeester E., Grosveld F., Gribnau J. (2008). X inactivation counting and choice is a stochastic process: evidence for involvement of an X-linked activator. Cell.

[25] Lyon M.F. (1971). Possible mechanisms of X chromosome inactivation. Nat N Biol.

[26] Barros de Andrade E., Sousa L., Jonkers I., Syx L., Dunkel I., Chaumeil J., Picard C., Foret B., Chen C.-J., Lis J.T., Heard E. (2019). Kinetics of Xist-induced gene silencing can be predicted from combinations of epigenetic and genomic features. Genome Res.

[27] Sun S., Del Rosario B.C., Szanto A., Ogawa Y., Jeon Y., Lee J.T. (2013). Jpx RNA activates Xist by evicting CTCF. Cell.

[28] Jonkers I., Barakat T.S., Achame E.M., Monkhorst K., Kenter A., Rentmeester E., Grosveld F., Grootegoed J.A., Gribnau J. (2009). RNF12 is an X-Encoded dose-dependent activator of X chromosome inactivation. Cell.

[29] Gontan C., Achame E.M., Demmers J., Barakat T.S., Rentmeester E., van IJcken W., Grootegoed J.A., Gribnau J. (2012). RNF12 initiates X-chromosome inactivation by targeting REX1 for degradation. Nature.

[30] Shin J., Bossenz M., Chung Y., Ma H., Byron M., Taniguchi-Ishigaki N., Zhu X., Jiao B., Hall L.L., Green M.R. (2010). Maternal Rnf12/RLIM is required for imprinted X-chromosome inactivation in mice. Nature.

[31] Shin J., Wallingford M.C., Gallant J., Marcho C., Jiao B., Byron M., Bossenz M., Lawrence J.B., Jones S.N., Mager J. (2014). RLIM is dispensable for X-chromosome inactivation in the mouse embryonic epiblast. Nature.

[32] Gontan C., Mira-Bontenbal H., Magaraki A., Dupont C., Barakat T.S., Rentmeester E., Demmers J., Gribnau J. (2018). REX1 is the critical target of RNF12 in imprinted X chromosome inactivation in mice. Nat Commun.

[33] Barakat T.S., Gunhanlar N., Pardo C.G., Achame E.M., Ghazvini M., Boers R., Kenter A., Rentmeester E., Grootegoed J.A., Gribnau J. (2011). RNF12 activates Xist and is essential for X chromosome inactivation. PLoS Genet.

[34] Tian D., Sun S., Lee J.T. (2010). The long noncoding RNA, Jpx, is a molecular switch for X chromosome inactivation. Cell.

[35••] Gjaltema R.A.F., Schwämmle T., Kautz P., Robson M., Schöpflin R., Ravid Lustig L., Brandenburg L., Dunkel I., Vechiatto C., Ntini E. (2022). Distal and proximal cis-regulatory elements sense X chromosome dosage and developmental state at the Xist locus. Mol Cell.

[36] Collombet S., Ranisavljevic N., Nagano T., Varnai C., Shisode T., Leung W., Piolot T., Galupa R., Borensztein M., Servant N. (2020). Parental-to-embryo switch of chromosome organization in early embryogenesis. Nature.

[37] Samanta M.K., Gayen S., Harris C., Maclary E., Murata-Nakamura Y., Malcore R.M., Porter R.S., Garay P.M., Vallianatos C.N., Samollow P.B. (2022). Activation of Xist by an evolutionarily conserved function of KDM5C demethylase. Nat Commun.

[38] Navarro P., Chambers I., Karwacki-Neisius V., Chureau C., Morey C., Rougeulle C., Avner P. (2008). Molecular coupling of Xist regulation and pluripotency. Science.

[39] Donohoe M.E., Silva S.S., Pinter S.F., Xu N., Lee J.T. (2009). The pluripotency factor Oct4 interacts with Ctcf and also controls X-chromosome pairing and counting. Nature.

[40] Payer B., Rosenberg M., Yamaji M., Yabuta Y., Koyanagi-Aoi M., Hayashi K., Yamanaka S., Saitou M., Lee J.T. (2013). Tsix RNA and the germline factor, PRDM14, link X reactivation and stem cell reprogramming. Mol Cell.

[41] Minkovsky A., Barakat T.S., Sellami N., Chin M.H., Gunhanlar N., Gribnau J., Plath K. (2013). The pluripotency factor-bound intron 1 of Xist is dispensable for X chromosome inactivation and reactivation in vitro and in vivo. Cell Rep.

[42] Buecker C., Srinivasan R., Wu Z., Calo E., Acampora D., Faial T., Simeone A., Tan M., Swigut T., Wysocka J. (2014). Reorganization of enhancer patterns in transition from naive to primed pluripotency. Cell Stem Cell.

[43] Delbridge A.R.D., Kueh A.J., Ke F., Zamudio N.M., El-Saafin F., Jansz N., Wang G.-Y., Iminitoff M., Beck T., Haupt S. (2019). Loss of p53 causes stochastic aberrant X-chromosome inactivation and female-specific neural tube defects. Cell Rep.

[44] Ravid Lustig L., Sampath Kumar A., Schwämmle T., Dunkel I., Noviello G., Weigert R., Pacini G., Buschow R., Ghauri A., Stoetzel M. (2022). GATA transcription factors drive initial Xist upregulation after fertilization through direct activation of a distal enhancer element. BioRxiv.

[45] Makhlouf M., Ouimette J.-F., Oldfield A., Navarro P., Neuillet D., Rougeulle C. (2014). A prominent and conserved role for YY1 in Xist transcriptional activation. Nat Commun.

[46•] Enervald E., Powell L.M., Boteva L., Foti R., Blanes Ruiz N., Kibar G., Piszczek A., Cavaleri F., Vingron M., Cerase A. (2021). RIF1 and KAP1 differentially regulate the choice of inactive versus active X chromosomes. EMBO J.

[47] Klein K.N., Zhao P.A., Lyu X., Sasaki T., Bartlett D.A., Singh A.M., Tasan I., Zhang M., Watts L.P., Hiraga S.-I. (2021). Replication timing maintains the global epigenetic state in human cells. Science.

[48] Cerase A., Young A.N., Ruiz N.B., Buness A., Sant G.M., Arnold M., Di Giacomo M., Ascolani M., Kumar M., Hierholzer A. (2021). Chd8 regulates X chromosome inactivation in mouse through fine-tuning control of Xist expression. Commun Biol.

[49] Aeby E., Lee H.-G., Lee Y.-W., Kriz A., Del Rosario B.C., Oh H.J., Boukhali M., Haas W., Lee J.T. (2020). Decapping enzyme 1A breaks X-chromosome symmetry by controlling Tsix elongation and RNA turnover. Nat Cell Biol.

[50] Robert-Finestra T., Tan B.F., Mira-Bontenbal H., Timmers E., Gontan C., Merzouk S., Giaimo B.D., Dossin F., van IJcken W.F.J., Martens J.W.M. (2021). SPEN is required for Xist upregulation during initiation of X chromosome inactivation. Nat Commun.

[51] Nora E.P., Lajoie B.R., Schulz E.G., Giorgetti L., Okamoto I., Servant N., Piolot T., van Berkum N.L., Meisig J., Sedat J. (2012). Spatial partitioning of the regulatory landscape of the X-inactivation centre. Nature.

[52•] Ohhata T., Yamazawa K., Miura-Kamio A., Takahashi S., Sakai S., Tamura Y., Uchida C., Kitagawa K., Niida H., Hiratani I. (2021). Dynamics of transcription-mediated conversion from euchromatin to facultative heterochromatin at the Xist promoter by Tsix. Cell Rep.

[53] Sado T., Hoki Y., Sasaki H. (2005). Tsix silences Xist through modification of chromatin structure. Dev Cell.

[54] Navarro P., Page D.R., Avner P., Rougeulle C. (2006). Tsix-mediated epigenetic switch of a CTCF-flanked region of the Xist promoter determines the Xist transcription program. Genes Dev.

[55] Ohhata T., Matsumoto M., Leeb M., Shibata S., Sakai S., Kitagawa K., Niida H., Kitagawa M., Wutz A. (2015). Histone H3 Lysine 36 trimethylation is established over the Xist promoter by antisense Tsix transcription and contributes to repressing Xist expression. Mol Cell Biol.

[56] Ogawa Y., Lee J.T. (2003). Xite, X-inactivation intergenic transcription elements that regulate the probability of choice. Mol Cell.

[57] Lee J.T., Davidow L.S., Warshawsky D. (1999). Tsix, a gene antisense to Xist at the X-inactivation centre. Nat Genet.

[58] Vigneau S., Augui S., Navarro P., Avner P., Clerc P. (2006). An essential role for the DXPas34 tandem repeat and Tsix transcription in the counting process of X chromosome inactivation. Proc Natl Acad Sci USA.

[59•] Galupa R., Nora E.P., Worsley-Hunt R., Picard C., Gard C., van Bemmel J.G., Servant N., Zhan Y., El Marjou F., Johanneau C. (2020). A conserved noncoding locus regulates random monoallelic Xist expression across a topological boundary. Mol Cell.

[60] Furlan G., Gutierrez Hernandez N., Huret C., Galupa R., van Bemmel J.G., Romito A., Heard E., Morey C., Rougeulle C. (2018). The ftx noncoding locus controls X chromosome inactivation independently of its RNA products. Mol Cell.

[61] Rosspopoff O., Cazottes E., Huret C., Loda A., Collier A.J., Casanova M., Rugg-Gunn P.J., Heard E., Ouimette J.-F., Rougeulle C. (2023). Species-specific regulation of XIST by the JPX/FTX orthologs. Nucleic Acids Res.

[62] van Bemmel J.G., Galupa R., Gard C., Servant N., Picard C., Davies J., Szempruch A.J., Zhan Y., Żylicz J.J., Nora E.P. (2019). The bipartite TAD organization of the X-inactivation center ensures opposing developmental regulation of Tsix and Xist. Nat Genet.

[63] Galupa R., Picard C., Servant N., Nora E.P., Zhan Y., van Bemmel J.G., El Marjou F., Johanneau C., Borensztein M., Ancelin K. (2022). Inversion of a topological domain leads to restricted changes in its gene expression and affects interdomain communication. Development.

[64] Rougeulle C., Chaumeil J., Sarma K., Allis C.D., Reinberg D., Avner P., Heard E. (2004). Differential histone H3 Lys-9 and Lys-27 methylation profiles on the X chromosome. Mol Cell Biol.

[65] Johnston C.M., Nesterova T.B., Formstone E.J., Newall A.E., Duthie S.M., Sheardown S.A., Brockdorff N. (1998). Developmentally regulated Xist promoter switch mediates initiation of X inactivation. Cell.

[66•] Oh H.J., Aguilar R., Kesner B., Lee H.-G., Kriz A.J., Chu H.-P., Lee J.T. (2021). Jpx RNA regulates CTCF anchor site selection and formation of chromosome loops. Cell.

[67] Hoki Y., Kimura N., Kanbayashi M., Amakawa Y., Ohhata T., Sasaki H., Sado T. (2009). A proximal conserved repeat in the Xist gene is essential as a genomic element for X-inactivation in mouse. Development.

[68] Royce-Tolland M.E., Andersen A.A., Koyfman H.R., Talbot D.J., Wutz A., Tonks I.D., Kay G.F., Panning B. (2010). The A-repeat links ASF/SF2-dependent Xist RNA processing with random choice during X inactivation. Nat Struct Mol Biol.

[69] Coker H., Wei G., Moindrot B., Mohammed S., Nesterova T., Brockdorff N. (2020). The role of the Xist 5′ m6A region and RBM15 in X chromosome inactivation. [version 1; peer review: 1 approved, 2 approved with reservations]. Wellcome Open Res.

[70] Szanto A., Aguilar R., Kesner B., Blum R., Wang D., Cifuentes-Rojas C., Del Rosario B.C., Kis-Toth K., Lee J.T. (2021). A disproportionate impact of G9a methyltransferase deficiency on the X chromosome. Genes Dev.

[71] Norris D.P., Patel D., Kay G.F., Penny G.D., Brockdorff N., Sheardown S.A., Rastan S. (1994). Evidence that random and imprinted Xist expression is controlled by preemptive methylation. Cell.

